# A revision of the millipede genus *Riukiupeltis* Verhoeff, 1939 (Diplopoda, Polydesmida, Paradoxosomatidae), with comments on the status of related species

**DOI:** 10.3897/zookeys.156.2009

**Published:** 2011-12-20

**Authors:** Anh D. Nguyen, Zoltán Korsós

**Affiliations:** 1 Institute of Ecology and Biological Resources, No18, Hoangquocviet Road, Caugiay District, Hanoi, Vietnam; 2 Department of Zoology, Hungarian Natural History Museum, Baross u. 13, H-1088 Budapest, Hungary; 3 Tropical Biosphere Research Center, University of the Ryukyus, Senbaru 1, Nishihara, Okinawa 903-0213, Japan

**Keywords:** Revision, *Riukiupeltis*, Paradoxosomatidae, Polydesmida, Vietnam, Japan

## Abstract

The East Asian millipede genus *Riukiupeltis* Verhoeff, 1939 is revised, and is restricted to a single species, *Riukiupeltis jamashinai* Verhoeff, 1939. Examination of the type specimens and freshly collected material from the Ryukyu Archipelago and Vietnam show that both subsequently allocated species, *Riukiupeltis uenoi* Murakami, 1975, and *Riukiupeltis falcatus* (originally *Haplogonosoma falcatum* Attems, 1953, reallocated by Jeekel 1968), do not belong to this genus; moreover, they are not even congeneric with each other. According to our morphological observations, including the gonopods, *Riukiupeltis uenoi* is closer to the widespread *Chamberlinius hualienensis* Wang, 1956, hence we propose the new combination *Chamberlinius uenoi* (Murakami, 1975), **comb. n.** *Riukiupeltis falcatus*, on the other hand, represents a separate, as yet monotypic, genus *Simplogonomorpha* **gen. n.**, distinct both from *Haplogonosoma* Brölemann, 1916 *sensu* Golovatch et al. (1995), and from Verhoeff’s original *Riukiupeltis*. Additionally, *Simplogonomorpha falcata* (Attems, 1953), **comb. n** is re-described here based on fresh material from Vietnam. A key and colour habitus-illustrations to all three species are also provided here.

## Introduction

The millipede genus *Riukiupeltis* was established for a single species *Riukiupeltis jamashinai* by Verhoeff, 1939 from the Ryukyu island Miyako, Japan (Riukiu and Mijako, in German). Jeekel (1968) referred to the genus in his monograph on the distribution of the family Paradoxosomatidae, and placed it in the tribe Tonkinosomatini. Moreover, he also tentatively allocated the species *Haplogonosoma falcatum* Attems, 1953, described from Xieng Khoang, Laos, to *Riukiupeltis*. Murakami (1975) described a new species *Riukiupeltis uenoi* from Sabichi-go Cave, Ishigaki-jima island, the Ryukyus. Therefore, altogether three species have been assigned to *Riukiupeltis*. The genus belongs to the tribe Chamberlinini, together with *Aponedyopus* Verhoeff, 1939, *Chamberlinius* Wang, 1956, *Geniculodesmus* Chen, Golovatch and Chang, 2008, and *Haplogonosoma* Brölemann, 1916 (Chen et al. 2011).

Following several discussions on the genus in the past (Jeekel 1968, Hoffman 1973, Murakami 1975, Chen et al. 2011), its status is still dubious, so our purpose here is to provide a revision of the genus based on fresh material and type specimens.

## Material and Methods

Fresh material of *Riukiupeltis falcatus* (Attems, 1953) and *Riukiupeltis uenoi* Murakami, 1975 was collected from Bi Doup National Park, Lam Dong province, Vietnam, and Iriomote-jima and Ishigaki-jima islands, the Ryukyu Archipelago, Japan, respectively. The type specimen of *Riukiupeltis jamashinai* was studied as a loan from the Bavarian State Collection of Zoology, Munich, Germany (BSCZ), whereas the holotype of *Riukiupeltis uenoi* was borrowed from the National Museum of Nature and Science, Tokyo (NMNS). Further material is shared between the University Museum (Fujukan) of the University of the Ryukyus, Okinawa (RUMF), the Hungarian Natural History Museum, Budapest (HNHM), and the Institute of Ecology and Biological Resources (IEBR), Hanoi, Vietnam. In addition, new material of *Riukiupeltis jamashinai* was identified in the collection by M. Shimojana, acquired in 1979 on Miyako-jima island.

Line drawings were made by using an Olympus SZX10 (ADN), and a Leica M125 (ZK) stereo microscope with drawing tube attached. SEM images were made by using a Hitachi S4800 scanning electron microscope. Colour photographs were taken by ZK using a Nikon D90 digital camera with macro lens and Leica microscope photo tube attached. The distribution map was generated using the software DIVA-GIS version 7.0.

## Taxonomic account

### Riukiupeltis

Verhoeff, 1939

http://species-id.net/wiki/Riukiupeltis

Riukiupeltis Verhoeff, 1939: Zoologischer Anzeiger 127 (5/6): 121–125 Type species: *Riukiupeltis jamashinai* Verhoeff, 1939, by monotypy
Riukiupeltis :– Attems 1940: Das Tierreich 70: 546–547.
Riukiupeltis :– Takakuwa 1954: *[Diplopoda of Japan]*: 51.
Riukiupeltis :– Jeekel 1968: *On the classification and geographical distribution of the family Paradoxosomatidae (Diplopoda, Polydesmida)*, Nederlandse Entomologische Vereiniging: 62, 76. (placed in the tribe Tonkinosomatini)

#### Diagnosis.

Gonofemorite strongly curved, distal part somewhat swollen and membraneous. Postfemoral region demarcated from femorite by obvious cingulum, and bent continuously forming almost a complete circle with femorite. Postfemoral regions consisting of a thick, strong and free solenomere, and an extremely short solenophore (= tibiotarsus).

#### Type species.

*Riukiupeltis jamashinai* Verhoeff, 1939

#### Remarks.

This genus is relatively close to the genus *Chamberlinius* Wang, 1956, however, it definitely differs in gonopod conformation: femorite without lamina; solenophore very short, thick, and without any basal processes.

### Riukiupeltis jamashinai

Verhoeff, 1939

http://species-id.net/wiki/Riukiupeltis_jamashinai

**Figure 1.**
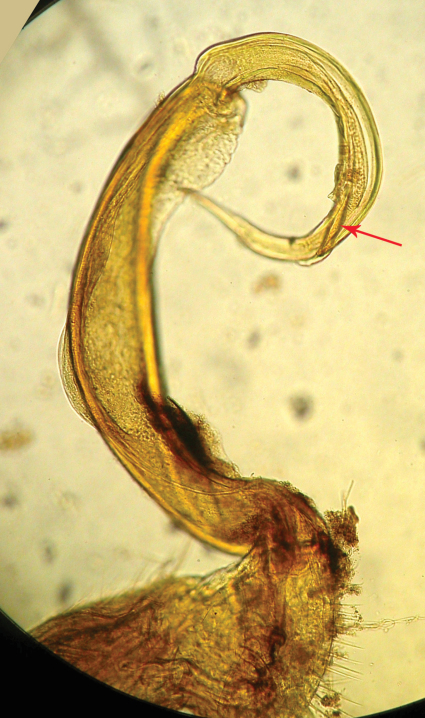
*Riukiupeltis jamashinai* Verhoeff, 1939, right gonopod preparation of holotype (slide Reg.-Nr. ZSMA20035204) (red arrow marks solenophore)

**Map 1.**
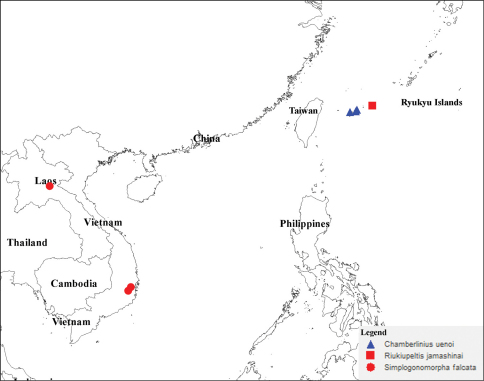
Distribution of three species *Chamberlinius uenoi* (Murakami, 1975) comb. n., *Riukiupeltis jamashinai* Verhoeff, 1939, and *Simplogonomorpha falcata* (Attems, 1953) comb. n.

Figs 1 2 6A 7A 8A Map 1

Riukiupeltis jamashinai Verhoeff, 1939: Zoologischer Anzeiger, 127 (5/6): 125, figs 8–9.
Riukiupeltis jamashinai :– Attems 1940: Das Tierreich 70: 547, fig. 693.
Riukiupeltis jamashinai :– Takakuwa 1954: *[Diplopoda of Japan]*, 52, figs 51–52.
Riukiupeltis jamashinai :– Jeekel 1968: *On the classification and geographical distribution of the family Paradoxosomatidae (Diplopoda, Polydesmida)*, Nederlandse Entomologische Vereiniging: 76.
Riukiupeltis jamashinai :– Nakamura and Korsós 2010: Acta Arachnologica 59(2): 82.

#### Material studied.

Holotype male, in fragments – only 11 segments in 5 pieces – with segments around gonopods missing, Reg.-Nr. ZSMA20052252, and two slides with gonopods, Reg.-Nr. ZSMA20035204, and legpairs 1–7, Reg.-Nr. ZSMA20035205 (all BSCZ).

New records: 2 males, 2 females, Japan, Ryukyu Archipelago, Miyako-jima Island, Rinko-abu (cave), 21 August 1979; 8 males, 2 females, Japan, Ryukyu Archipelago, Miyako-jima Island, Fukumine-no-kara (cave), 25 August 1979; 1 male, 1 female, 1 juv., 1 fragment, Japan, Ryukyu Archipelago, Miyako-jima Island, Nishibe zuzaga (cave), 26 August 1979, all leg. M. Shimojana (in the collection of M. Shimojana, Okinawa).

#### Distribution.

Japan, Ryukyu Archipelago, Miyako-jima island.

#### Remarks.

**Figure 2.**
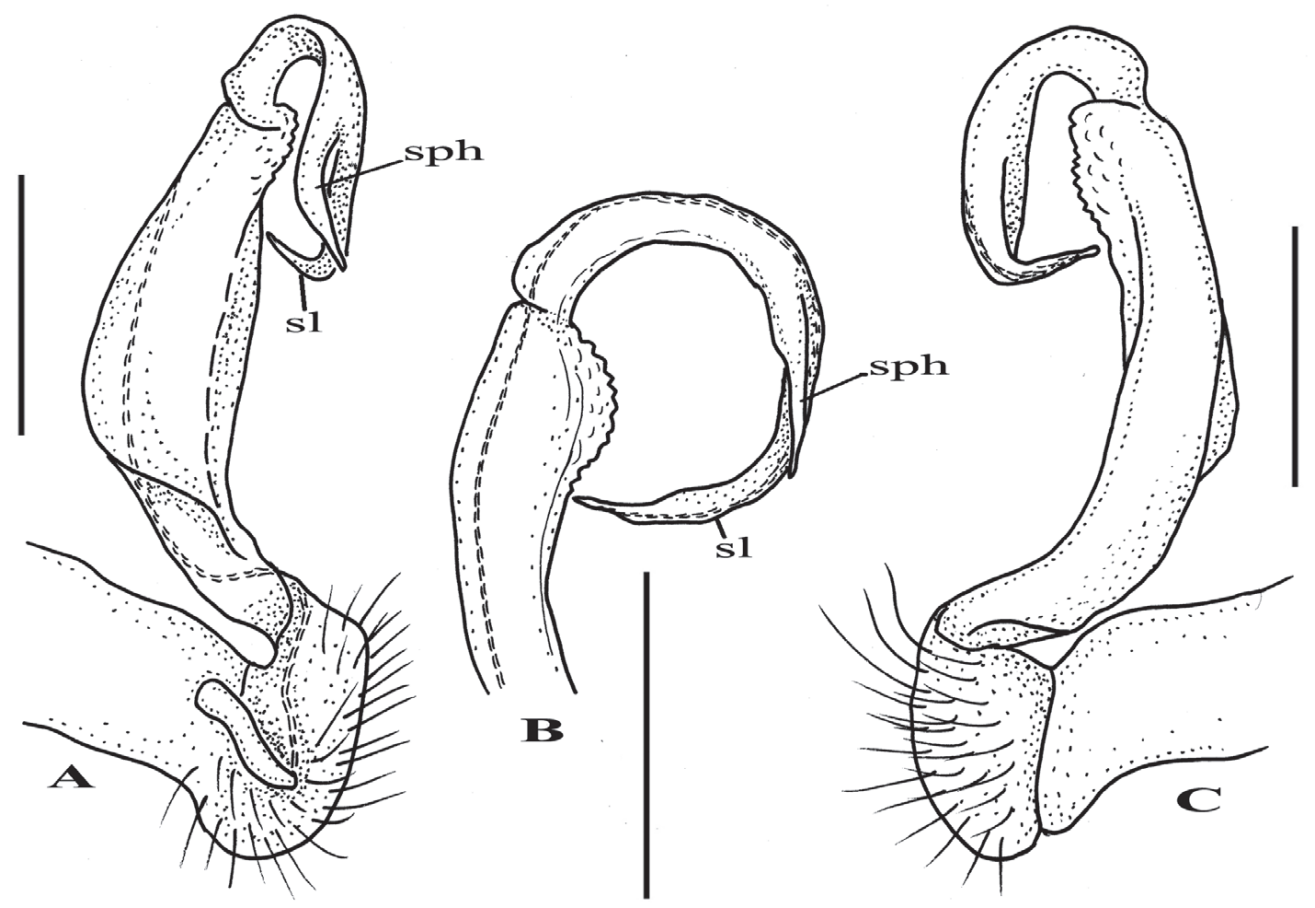
*Riukiupeltis jamashinai* Verhoeff, 1939, left gonopod of male from Fukumine-no-kara cave, Miyako-jima Island, 25 August 1979, leg. M. Shimojana **A** mesal view **B** dorsal view **C** ventro-lateral view (*sl* = solenomere, *sph* = solenophore or tibiotarsus). Scale bars = 0.5 mm.

Although after the description of *Riukiupeltis jamashinai* in 1939, Jeekel (1968) and Hoffman (1973) commented that gonopod tibiotarsus is missing in this species, Verhoeff’s line drawing clearly shows it as depicted from the slide preparation (Fig. 1). Re-examining the type specimen and the slide of the gonopod, as well as studying newly identified specimens found in Shimojana’s collection, we are able to confirm that a gonopod tibiotarsus (=solenophore, ***sph*** in Fig. 2) is present, although it is small and closely attached to solenomere (***sl***in Fig. 2).

### Chamberlinius uenoi

(Murakami, 1975) comb. n.

http://species-id.net/wiki/Chamberlinius_uenoi

**Figure 3.**
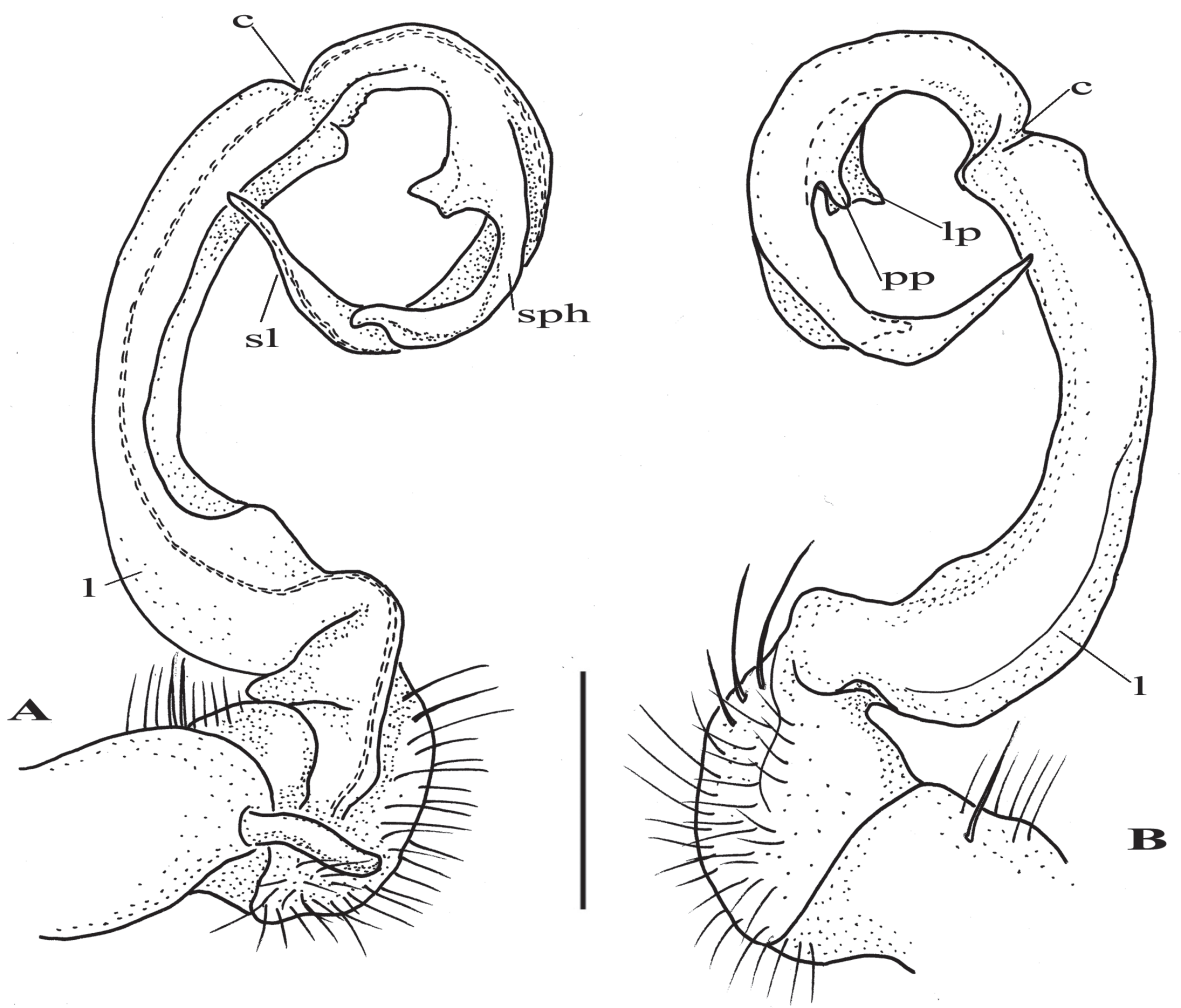
*Chamberlinius uenoi* (Murakami, 1975) comb. n.**,** left gonopod of male Mt. Banna-dake, Ishigaki-jima Island **A** dorso-mesal view **B** lateral view. (*l* = lamina, *c* = cingulum, *sl* = solenomere, *sph* = solenophore or tibiotarsal process, *lp* = laminar process, *pp* = pointed process)). Scale bar = 0.5 mm.

Figs 3 6C 7C 8C Map 1

Riukiupeltis uenoi Murakami, 1975: Bulletin of the National Science Museum, Tokyo, {A}1(2): 105–107, fig. 9
Riukiupeltis uenoi :– Nakamura and Korsós 2010: Acta Arachnologica 59(2): 82

#### Material studied.

Holotype male, NSMT-My 358, “Japan, Okinawa Pref., Is. Ishigaki-jima, Ibaruma, Sabichi-go Cave, 31 July 1973, coll. by S. Uéno” (NMNS).

New records: 2 males, 1 female, Japan, Ryukyu Archipelago, Yaeyama Island Group, Ishigaki-jima Island, Banna-dake, secondary forest, N24.3859°, E124.1651°, 30 August 2009, leg. Z. Korsós & Y. Nakamura (RUMF); 1 male, 2 females, Japan, Southern Ryukyus, Yaeyama Group, Iriomote-jima Island, Funaura, around university research station, N24.3929°, E123.7913°, secondary forest, 18 January 2011, leg. Z. Korsós (RUMF); 1 male, 1 female, Japan, Southern Ryukyus, Yaeyama Group, Iriomote-jima Island, Mihara, along Aira river, primary forest, N24.3400°, E123.9137°, in decaying log, 12 m a.s.l., 19 January 2011, leg. Z. Korsós (HNHM).

#### Distribution

**.** Japan, Ryukyu Archipelago, Yaeyama Group, Ishigaki-jima and Iriomote-jima islands.

#### Remarks.

Murakami (1975) when describing the species commented that the terminal portion of the gonopod is more complicated than that of *Riukiupeltis jamashinai* Verhoeff, 1939. He also agreed with Verhoeff, 1939 in its configuration, and placed his species in *Riukiupeltis*. However, the species *uenoi*, in fact, differs largely from the type species *Riukiupeltis jamashinai* in gonopod conformation.

After studying the type and freshly collected specimens, it became clear that the species *uenoi* is strongly different from *Riukiupeltis jamashinai* in its gonofemorite having a long lamina ***l***, and a longer solenophore with basal processes ***pp*** and ***lp*** (Fig 3). We found that this species is more similar to *Chamberlinius hualienensis* Wang, 1956. Both *Chamberlinius hualienensis* and *Chamberlinius uenoi* comb. n. have well-developed paraterga (Figs 7A–B), large, slender and strongly concave gonofemorite, with a lamina at the mesal side (***l*** in Fig. 3). Postfemoral region is demarcated from femorite by obvious cingulum (***c*** in Fig. 3), and includes a long and large solenomere (***sl***) reaching femur, and a shorter solenophore (***sph***) with a basal lobe. However, the two species differ from each other in the length of the postfemoral processes, by the shape of the small basal processes on the solenophore, and by live colouration. The dark brown, transversal metatergal bands in *Chamberlinius uenoi* comb. n. are not divided by a median light brown longitudinal line (Figs 7C, 8C) as in *Chamberlinius hualienensis* (Figs 7B, 8B). Moreover, *Chamberlinius uenoi* comb. n. is strictly confined to undisturbed, natural evergreen broadleaf forests, and can only be found deep in decaying dead wood, whereas *Chamberlinius hualienensis* has a strong tendency for being synanthropic, and dispersed in large numbers onto many islands (especially in the southern part of Japan) by human activities.

### Simplogonomorpha gen. n.

urn:lsid:zoobank.org:act:878B9422-C9BB-462E-87A5-4A2610BB5B52

http://species-id.net/wiki/Simplogonomorpha

#### Type species.

*Haplogonosoma falcatum* Attems, 1953, by present designation

#### Diagnosis.

This genus, *Simplogonomorpha* gen. n. can be distinguished from other genera within the tribe Chamberlinini Wang, 1956 (as defined by Chen et al. 2011) by the following characters: paraterga modestly developed, gonopod very simple, gonotelopodite tapering and distally curved down as much as forming U-shape or an almost complete circle. Solenomere very simple, thick, but slender, and strongly curved down. Solenophore (= gonopod tibiotarsus) absent.

#### Etymology.

A feminine noun to emphasize the simple gonopod conformation.

#### Remarks.

Jeekel (1968) in his classification of the family Paradoxosomatidae stated that “It appears that in *Riukiupeltis* the gonopod tiobiotarsus is also completely lost, although Verhoeff was of a different opinion when he described *jamashinai*”. He believed that the gonopod tibiotarsus (= solenophore) was lost in *Riukiupeltis*, so he transferred Attems’s species *Haplogonosoma falcatum* to this genus.

In fact, the solenophore of *Riukiupeltis jamashinai* still exists, although short, and somewhat hidden next to the solenomere, whereas solenophore of *Simplogonomorpha* gen. n. is totally missing. A comparison of genera in the tribe Chamberlinini is provided in Table 1.

**Table 1.** Comparison of genera of Chamberlinini

No.	Characters	Riukiupeltis	Chamberlinius	Simplogonomorpha	Aponedyopus	Haplogonosoma	Geniculodesmus
1	Paraterga	Well-developed	Well-developed	Modestly-developed	Poorly-developed	Poorly-developed	Poorly-developed
2	Sternal process between coxae 4	Missing	Missing	Two separate cones	A single or bifid process	A single process	Linguiform
Gonopod conformation							
3	Coxa	Long, cylindrical but not thick	Long, thick and subcylindrical	Thick and subcylindrical	Long and subcylindrical	Long and subcylindrical	Long and subcylindrical
4	Femorite	Long and slender, with a rugose membranous lamina at distal part of lateral side	Especially long, slender, simple without any outgrowths, however, sometimes, with a lamina at mesal side	Especially long, but simple and slender, devoid of any lobes or lamina.	Long, but broadened parabasally at dorsal side, without any lamina or processes	Long, slender and erected, a little bit broadened distally, devoid of any modifications	Long, slightly curved, not broadened at base
5	Postfemoral part	Demarcated by cingulum	Demarcated by cingulum	Demarcated by cingulum	Demarcated by obvious sulcus at lateral side	Set off by oblique sulcus at lateral side, apical part with a big tooth.	absent
6	Solenomere	Long, thick and simple, not sheathed by solenophore	Long, thick and simple, not sheathed by solenophore	Thick, but slender, and strongly curved downwards	Flagiliform, almost completely sheathed by solenophore, sometimes, only tip exposed	Long, slender and coiled, subfiliform	Long, flagiliform, almost sheathed by solenophore
7	Solenophore	Very short, simple, without any additional processes	As long as solenomere, with a basal dentiform outgrowth	Completely missing	Base with a small, obvious lobe. Tip with bifid lobe	Laminate, coiled, and as long as solenomere	Long and coiled, with a process at base

### Simplogonomorpha falcata

(Attems, 1953) comb. n.

http://species-id.net/wiki/Simplogonomorpha_falcata

Figs 4 5 7D Map. 1

Haplogonosoma falcatum Attems, 1953: Mémoires du Muséum National d’Histoire Naturelle, Paris {N. S., Sér. A, Zool.}, 5(3): 177, figs 81–82.
Riukiupeltis falcatus :– Jeekel 1968: *On the classification and geographical distribution of the family Paradoxosomatidae (Diplopoda, Polydesmida)*, Nederlandse Entomologische Vereiniging: 62.

#### Material studied.

3 males, 2 females, (IEBR-166), Vietnam, Lam Dong prov., Bi Doup-Nui Ba National Park, corn field, 1400m a.s.l., pitfall traps, 2–9 April 2008, leg. Anh. D. Nguyen; 1 male, 1 female, 1 juvenile, (IEBR-167), same locality, grasslands, 1400m a.s.l., pitfall traps, 2–9 April 2008, leg. Anh. D. Nguyen; 1 female, (IEBR-168), same locality, bushes near stream, 1400m a.s.l., pitfall traps, 25 April 2008, leg. Anh. D. Nguyen; 4 males, 2 females, (IEBR-169), same locality, evergreen forest, 1800m a.s.l., 25 March–23 April 2008, leg. Anh. D. Nguyen;1 male (IEBR-125), Vietnam, Khanh Hoa Prov., Hon Ba Mts., 1300–1500m a.s.l., primary forest, 15–24 April 2006, leg. Anh. D. Nguyen; 1 male, 1 female, (HNHM), same data as sample IEBR-125.

#### Description. 

*Head*: yellowish-brown to blackish brown, a slightly paler toward labrum. Epicranial suture distinct, obviously deep.

*Antennae:* short and stout, yellowish-brown to blackish brown. Length of antennomere 2 subequal to that of antennomere 3, 4 or 5. Antennomere 6 shorter and claviform.

*Body:* yellowish-brown to blackish brown on terga, paler on paraterga and pleura. Body parallel-sided on somites 5–17, thereafter gradually tapering.

Surface of metaterga general fine in posterior part, but with small oblique or longitudinal rugulose in anteriormost part. Stricture dividing pro- and metazona deep, obvious and beaded. Prozona surface shagreened with fine microgranulation**.**

Transverse sulcus on metaterga starting from somite 5 and more evident on subsequent somites. Metaterga with a row of 2+2 setae in pre-sulcus part. Axial line vague.

Paraterga not well-developed, small, look like small keels in poreless segments, but more developed in pore-bearing somites. Ozopore located on lateral side, near tip of angular paraterga of segments 5, 7, 9–10, 12–13 and 15–19.

Pleura shagreened with fine microgranulation. Pleurosternal carinae rather well-developed in anteriormost segments, gradually decreasing posteriorly.

Epiproct truncated and curved down ventrad, with 4 strong setae on tip. Anal valves sub-semicircular with 1+1 long setae, and a deep emargination inbetween. Hypoproct trapeziform, with 1+1 setae.

*Legs:* yellow to yellowish brown, short and stout. Tarsal brushes present until legpair 10, sparsely until legpair 16, and completely missing thereafter.

*Sterna:* normal, sparsely setose, with two large tubercles between coxae 4.

*Male gonopod:* very simple, hook-like in dorsal view. Coxa subcylindrical, distoventral part sparsely setose. Prefemoral part usually densely setose, with evident demarcation from both femorite and coxa. Femorite slender, much longer than coxite and a little curved down distally, separated from postfemoral part by an evident, subtransverse sulcus laterally and mesally. Solenomere simple, strongly curved down, slender, and tapering at tip. Tibiotarsus totally absent.

Prostatic groove runs mesally along femorite, distolaterad, and turns to lateral side, then running mesally, and ending at tip of solenomere.

**Figure 4.**
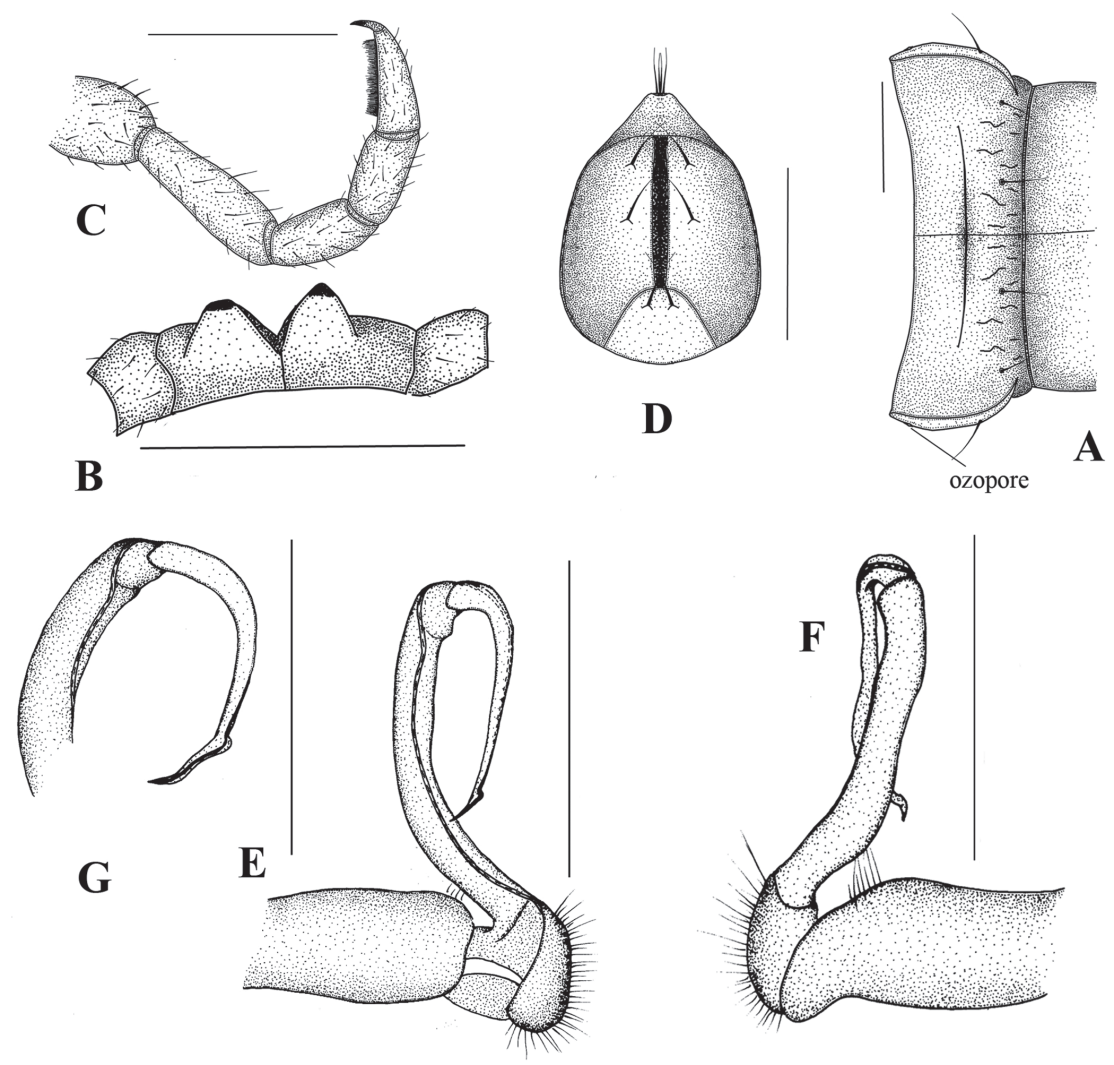
*Simplogonomorpha falcata* (Attems, 1953) comb. n. from Vietnam, BiDoup National Park **A** 10^th^ body segment, dorsal view **B** sternal processes between 4^th^ coxae, posteriovenral view **C** leg 10, lateral view **D** telson, ventral view **E–G** right gonopod **E** mesal **F** lateral, and **G** subdorsal view. Scale bar = 1 mm.

**Figure 5.**
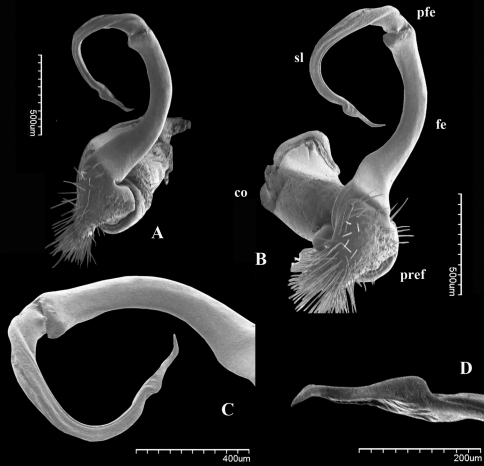
*Simplogonomorpha falcata* (Attems, 1953) comb. n. from Vietnam, BiDoup National Park, right gonopod: ventral **A** and mesoventral view **B, C** Tip of gonopod, ventral view **D**.

**Figure 6.**
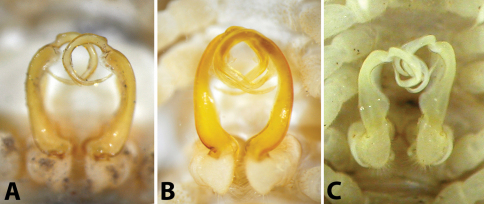
Male gonopods *in situ*, ventral view: **A** *Riukiupeltis jamashinai* (Miyako-jima Island) **B** *Chamberlinius hualienensis* (Okinawa-jima Island) **C** *Chamberlinius uenoi* comb. n.(Ishigaki-jima Island).

**Figure 7.**
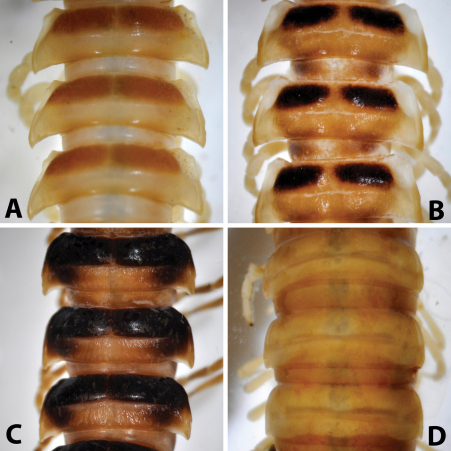
Midbody (11^th^-13^th^) segments, dorsal view: **A** *Riukiupeltis jamashinai* (Miyako-jima Island) **B** *Chamberlinius hualienensis* (Okinawa-jima Island) **C** *Chamberlinius uenoi* comb. n.(Ishigaki-jima Island) and **D** *Simplogonomorpha falcata* comb. n. (Vietnam, Hon Ba Mts.)

**Figure 8.**
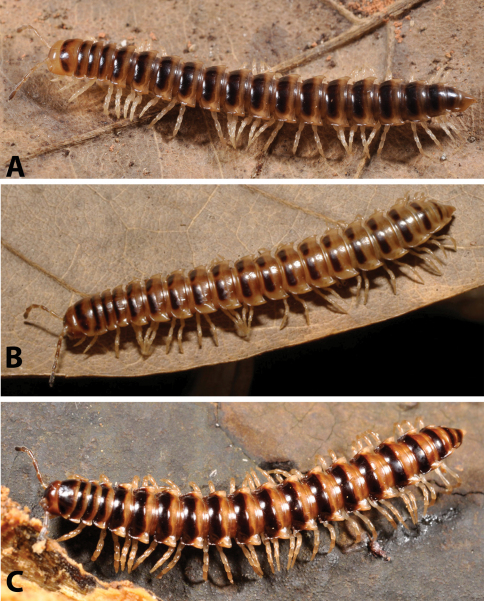
Habitus of millipedes: **A** *Riukiupeltis jamashinai* (live from Miyako-jima Island) **B** *Chamberlinius hualienensis* (live on Okinawa-jima Island) and **C** *Chamberlinius uenoi* comb. n.(live on Iriomote-jima Island).

#### Distribution.

Vietnam, Lam Dong province, Bi Doup-Nui Ba National Park; Khanh Hoa province, Hon Ba Mountain; Laos PDR, Xieng Khoang

#### Remarks.

New material does not much differ from Attems’s description. Only minor difference is the presence of two separate cones between coxae 4 instead of only one small conal process in Attems’s description. Recently, Chen et al. (2011) also published an illustration of gonopods of *Haplogonosoma falcatum* collected from the same locality, BiDoup National Park, Vietnam. Our material here fits well with their unevaluated illustration.

##### Key to representatives to all three genera (based on male characters)

**Table:** 

1	Paraterga very weak, body looks almost cylindrical, colour uniformly light brown-yellowish. Gonofemorite very long and slender, without any modifications or processes. Postfemoral region consisting only a simple, strong, long solenomere. Solenophore totally absent (Figs 4–5)	*Simplogonomorpha*
–	Paraterga well-developed with strong, triangular, posterio-lateral processes. Dorsal metazona colouration divided into dark brown anterior and much lighter posterior half, transversely separated by a deep sulcus	2
2	Gonofemorite large, slender and strongly concave, with a lamina on the mesal side. Postfemoral region with a long, large solenomere and a shorter solenophore, the latter basally with two processes, a laminar mesal (*lp*) and a more pointed lateral one (*pp*) (Fig. 3B)	*Chamberlinius*
–	Gonofemorite only slightly curved, with a weak dorsal lamina, distal part swollen and membraneous. Postfemoral region consisting a thick, strong, free solenomere, and a short, somewhat hidden solenophore (Fig. 2)	*Riukiupeltis*

In the key above, *Riukiupeltis* and *Simplogonomorpha* are represented by only one species each (*jamashinai* and *falcata*, respectively). *Chamberlinius*, on the other hand, includes at present five species: *Chamberlinius hualienensis*, *Chamberlinius piceofasciatus*, *Chamberlinius pessior*, *Chamberlinius sublaevus* (all keyed already by Chen et al. 2011), and *Chamberlinius uenoi*, as added here.

## Conclusion

As a result of our character comparisons, the taxonomic status of the following three species: *Riukiupeltis jamashinai* Verhoeff, 1939, *Riukiupeltis uenoi* Murakami, 1975 and *Riukiupeltis falcatus* (Attems, 1953) has been clarified. Based on significant morphological differences in body shape and gonopod structure, they all belong in three different genera. *Riukiupeltis jamashinai* having priority is maintained as such, whereas *uenoi* is transferred to *Chamberlinius* Wang, 1956, and a new genus, *Simplogonomorpha* gen. n., is erected to accommodate *falcatum*.

## Supplementary Material

XML Treatment for Riukiupeltis

XML Treatment for Riukiupeltis jamashinai

XML Treatment for Chamberlinius uenoi

XML Treatment for Simplogonomorpha

XML Treatment for Simplogonomorpha falcata
